# Intensive Communicative Therapy Reduces Symptoms of Depression in Chronic Nonfluent Aphasia

**DOI:** 10.1177/1545968317744275

**Published:** 2017-12-01

**Authors:** Bettina Mohr, Benjamin Stahl, Marcelo L. Berthier, Friedemann Pulvermüller

**Affiliations:** 1 Charité Universitätsmedizin Berlin, Department of Psychiatry, Berlin, Germany; 2 Charité Universitätsmedizin Berlin, Department of Neurology, Berlin, Germany; 3 Max Planck Institute for Human Cognitive and Brain Sciences, Leipzig, Germany; 4 Universität Greifswald, Department of Neurology, Germany; 5 Freie Universität Berlin, Brain Language Laboratory, Department of Philosophy and Humanities, Berlin, Germany; 6 University of Malaga and Instituto de Investigaciones Biomédicas de Málaga (IBIMA), Unit of Cognitive Neurology and Aphasia, Centro de Investigaciones Medico-Sanitarias (CIMES), Malaga, Spain; 7 Cathedra ARPA of Aphasia, Malaga, Spain; 8 Berlin School of Mind and Brain, Humboldt University, Berlin, Germany; 9 Einstein Center for Neurosciences, Berlin, Germany

**Keywords:** aphasia, depression, communication, neurological rehabilitation, language therapy

## Abstract

*Background.* Patients with brain lesions and resultant chronic aphasia frequently suffer from depression. However, no effective interventions are available to target neuropsychiatric symptoms in patients with aphasia who have severe language and communication deficits. *Objective.* The present study aimed to investigate the efficacy of 2 different methods of speech and language therapy in reducing symptoms of depression in aphasia on the Beck Depression Inventory (BDI) using secondary analysis (BILAT-1 trial). *Methods.* In a crossover randomized controlled trial, 18 participants with chronic nonfluent aphasia following left-hemispheric brain lesions were assigned to 2 consecutive treatments: (1) intensive language-action therapy (ILAT), emphasizing communicative language use in social interaction, and (2) intensive naming therapy (INT), an utterance-centered standard method. Patients were randomly assigned to 2 groups, receiving both treatments in counterbalanced order. Both interventions were applied for 3.5 hours daily over a period of 6 consecutive working days. Outcome measures included depression scores on the BDI and a clinical language test (Aachen Aphasia Test). *Results.* Patients showed a significant decrease in symptoms of depression after ILAT but not after INT, which paralleled changes on clinical language tests. Treatment-induced decreases in depression scores persisted when controlling for individual changes in language performance. *Conclusions.* Intensive training of behaviorally relevant verbal communication in social interaction might help reduce symptoms of depression in patients with chronic nonfluent aphasia.

## Introduction

Aphasia is a common neurological condition usually resulting from focal damage to perisylvian regions in the language-dominant left hemisphere (LH). Chronic language, communication, and motor impairments associated with aphasia often have a detrimental effect on the quality of life and psychological well-being of patients.^1,2^ In fact, many participants with aphasia suffer from psychiatric disorders, particularly poststroke depression (PSD).^3^ The annual prevalence rate of PSD ranges from 17% to 38%.^4^ Clinical symptoms of depression such as low mood, reduced drive, fatigue, and cognitive problems frequently persist in chronic aphasia^5-7^ and have a negative impact on functional recovery.^8,9^ Unfortunately, depression in aphasia often remains undiagnosed and untreated.^10^ Only few studies on PSD have so far included aphasic patients,^11^ partly because it has been argued that communication deficits preclude the diagnosis of PSD in this patient group. Although diagnostic instruments such as the Structured Clinical Interview for Diagnostic and Statistical Manual of Mental Disorders^12^ cannot be used for all participants with aphasia, alternative methods for diagnosis of affective disorders can be applied.^5^

To date, treatment of PSD has largely focused on drug treatment with antidepressants (mostly selective serotonin reuptake inhibitors [SSRIs]). In a Cochrane review, including 16 randomized controlled trials (RCTs), the effectiveness of pharmacological and psychological interventions in treating patients with PSD was evaluated. The evaluation showed that drug treatment was minimally effective over placebo and that psychological intervention alone was not effective in ameliorating symptoms of PSD.^13^

Psychological therapies in PSD have used psychoeducative interventions^14,15^ as well as cognitive behavioral therapy (CBT), a highly effective method to treat major depressive disorders.^16^ However, none of these methods was effective in PSD patients, and importantly, participants with aphasia were excluded from most previous pharmacological and nonpharmacological studies of PSD. Therefore, it is important and timely to find appropriate treatments for the clinical group of aphasics with PSD.

The study aimed to test (1) whether speech-language therapy tailored at improving communicative language skills, can help improve language deficits in patients with aphasia. As a secondary aim, we tested (2) whether aphasia therapy can ameliorate symptoms of depression and whether (3) improvements in depressive symptoms are associated with specific treatment methods. Crucially, it was investigated (4) whether changes in depression scores are associated with language improvements after treatment. To our knowledge, the present study is the first RCT (BILAT-1 trial) to address treatment effects on depression specifically in patients with aphasia.

Individuals with chronic aphasia participated in two types of intensive language therapy: intensive language-action therapy (ILAT),^17-19^ an extended form of constraint-induced aphasia therapy (CIAT),^20-22^ which emphasizes the training of language as a tool for communication in social-interactive settings, and a more traditional speech and language therapy method, naming therapy,^23^ which emphasizes utterance production to pictorial stimuli. Both methods made use of standard techniques known from CBT, including positive reinforcement and shaping. A crossover RCT was implemented in which each patient was assigned to one of two groups either receiving ILAT followed by intensive naming therapy (INT) or the reversed order. Several previous RCTs already demonstrated the efficacy of ILAT/CIAT^20,22^ and naming^23^ in improving language functions in participants with chronic aphasia (see also a recent Cochrane Review^24^). Furthermore, an ILAT-related increase in the amount of daily communicative activities has previously been reported.^20,25^ A recent RCT had shown better outcomes with ILAT as compared with INT.^26^ To specifically explore the potential effect of language rehabilitation on symptoms of depression, we here investigate changes in symptoms of depression in patients undergoing these two types of intensive language therapy. To assess symptoms of depression, we focused on a standard depression scale, the Beck Depression Inventory (BDI). We expected to find a reduction in symptoms of depression to be associated with therapy-induced language improvements. Moreover, we entertained the possibility of symptom improvements to be related to the specific type of language intervention.

## Methods

### Patients

A total of 18 patients (7 females) with chronic nonfluent aphasia participated in this study and were recruited from self-help groups by advertisements on web pages or were referred to us by local speech and language therapists. This sample size was based on a previous power analysis on a standardized aphasia test battery, the Aachen Aphasia Test (AAT; α = .05, 1 − β = 0.95; number of groups = 2; number of repeated measures = 3; estimated Cohen’s *f* = 0.4) derived from our previous study^20^ and equivalent to an increase of 2 points per training period on averaged T-scores on the AAT.^27^ Inclusion criteria for study participation were the following: (1) right-handedness according to the Edinburgh Handedness Inventory^28^ prior to disease onset, (2) native speakers of German, (3) no severe memory or auditory language comprehension deficits, and (4) nonfluent aphasia. Aphasia was assessed by the AAT.^29^ One patient was included following aphasia diagnosis based on records but was diagnosed as nonaphasic according to Token Test (AAT) criteria at study onset and was, therefore, subsequently excluded from all statistical analyses (although he completed both treatment intervals). Therefore, statistical analysis focused on the 17 individuals with confirmed aphasia. Nonverbal short-term memory was assessed by the Corsi Block Tapping Task^30^ and confirmed unimpaired short-term memory for all patients (mean score = 5.4; SD = 1.30). Physical disability was assessed by the Barthel Index/Activities of Daily Living Scale (ADL),^31^ which measures the (physical) independence during activities of daily living (higher scores indicate higher independence; maximum: 100; see Table 1).

**Table 1.** Clinical and Sociodemographic Patient Characteristics.^a^

Patient No. and Group	Age (years)	Sex	Education (School Years)	Aphasia Type and Severity	Disease Duration (years)	ADL/Barthel Index	Pretreatment AAT Score	SSRI or SNRI	Pretreatment BDI Score
1-1	46	F	13	Moderate-severe Broca’s aphasia	4.0	50	46	Yes	**15** *
3-1	49	M	10	Severe Broca’s aphasia	3.4	60	45	Yes	12
10-1	73	M	9	Global aphasia	5.0	35	39.5	Yes	8
11-1	39	F	10	Severe Broca’s aphasia	6.5	95	45.6	Yes	**33** *
12-1	49	F	10	Moderate Broca’s aphasia	1.4	100	49.3	Yes	**26** *
16-1	47	F	10	Mild Broca’s aphasia	20.4	100	61	No	12
17-1	37	F	13	Mild-moderate Broca’s aphasia	2.5	100	54.3	Yes	3
18-1	65	M	13	Moderate Broca’s aphasia	19.9	100	48.5	No	**27** *
4-2	41	F	13	Mild Broca’s aphasia	8.0	100	58.3	Yes	**51** **
5-2	49	M	13	Mild-moderate Broca’s aphasia	4.3	100	58.3	No	**50** **
6-2	54	M	13	Mild-moderate Broca’s aphasia	4.0	100	52.8	No	**14** *
7-2	35	F	9	Severe Broca’s aphasia	1.1	85	39	No	**15** *
8-2	32	M	9	Mild Broca’s aphasia	3.3	100	61.3	No	**18** *
9-2	62	M	12	Global aphasia	1.9	85	42.8	No	9
13-2	51	M	10	Moderate Broca’s aphasia	3.5	80	48.5	No	**53** **
14-2	63	M	10	Moderate Broca’s aphasia	2.5	90	50.8	No	11
15-2	66	M	9	Global aphasia	6.4	75	40.3	No	10
Mean	50.47		10.94		5.77	85.59	49.49		**21.59**
SD	11.95		1.71		5.71	19.99	7.29		**16.03**

Abbreviations: AAT, Aachen Aphasia Test; ADL, Activities of Daily Living Scale; BDI, Beck Depression Inventory; BDI-V, simplified 20-item German version of the original BDI; F, female; ILAT, intensive language-action therapy; INT, intensive naming therapy; M, male; SNRI, selective serotonin noradrenalin reuptake inhibitors; SSRI, selective serotonin reuptake inhibitors.

a Patients (1-18) were assigned either to group 1 with treatment order ILAT-INT (1) or to group 2 with treatment order: INT-ILAT (2). Higher AAT^29^ scores indicate better language performance. Aphasia type and severity was categorized by AAT scores, represented by mean T-scores for the following 4 subscales: token test, repetition, auditory comprehension, and naming. Range of BDI-V scores: minimum, 0; maximum, 60. Higher BDI^36^ scores indicate more severe symptoms of depression (0-13, no depression; *14-34, mild depression; **>35, moderate to severe depression). Clinically relevant BDI scores are in bold.

All patients presented with large fronto-temporo-parietal lesions in the LH; 15 patients had suffered a single cerebrovascular accident resulting in lesions in the left frontal, parietal, and temporal cortex and in adjacent subcortical areas. One patient had traumatic brain injury (patient 1-1) and another patient viral encephalopathy (patient 15-2) with lesions exclusively confined to the LH. Two clinical neuroscientists manually delineated and superimposed the precise locations of lesioned voxels in all patients using the software MRIcron.^32^ More data on patients’ lesion size and location have been reported previously.^26^ Seven patients (44,4%) received antidepressant medication (SSRIs and selective serotonin noradrenalin reuptake inhibitors); of these, 4 patients showed symptoms of mild to moderate depression (see Table 1) according to the BDI.^33^ Medication was not changed prior to commencement of the study, nor during the trial. Only patients in the chronic stage of the disease (>1 year post–disease onset) were included to prevent any effects related to spontaneous remission rather than to therapeutic intervention. Patients did not engage in any other (non–study-related) speech-language therapy during the RCT. Patient characteristics are presented in Table 1. The trial was approved by Charité University Medical School Ethics Committee, Berlin, Germany. Informed verbal and written consent was obtained from all patients.

### Study Design

**Figure 1.**
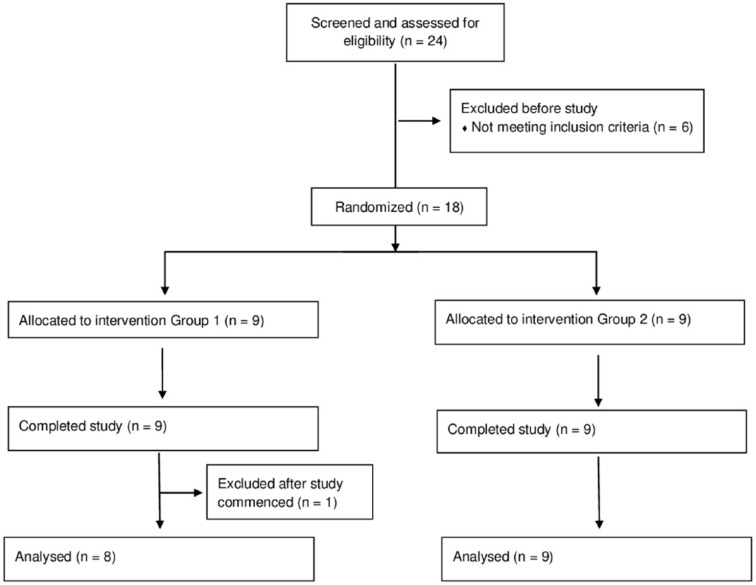
Consort flow diagram.

**Figure 2.**
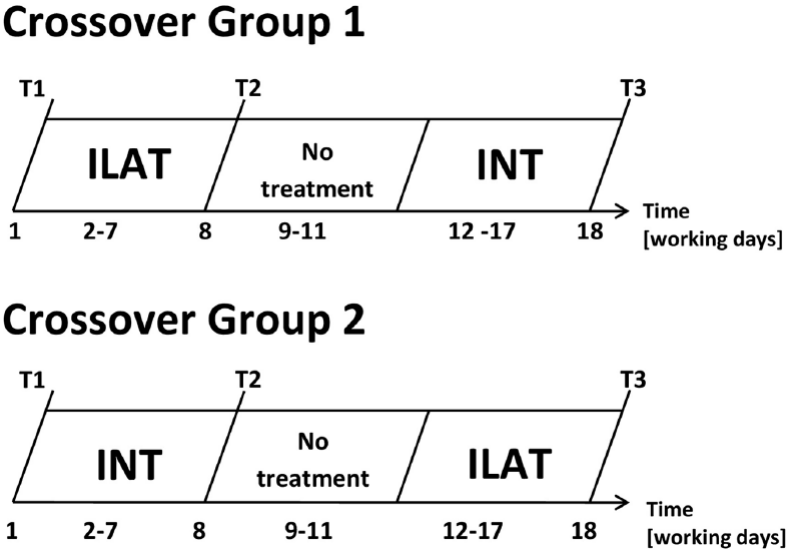
Study design showing the time course of treatment intervals and clinical assessment for both treatment groups. Patients were tested at 3 points in time: before treatment (T1), after the ﬁrst (T2) and after the second treatment (T3) interval. Abbreviations: ILAT, intensive language-action therapy; INT, intensive naming therapy.

In a crossover RCT, patients were randomly assigned to 1 of the two treatment groups by a computer-generated randomization program. Group allocation was performed by a person who alone had access to this list and who was not involved in recruitment, treatment, or assessment throughout this trial. This allocation procedure resulted in 9 patients per group (see Figure 1). In group 1, patients received ILAT prior to INT, whereas patients in group 2 engaged in INT, followed by ILAT. Both treatment methods are effective and established interventions to treat language deficits in aphasia.^20,23,24^ Each therapy method was applied with identical intensity for 3.5 hours per day (including breaks) and identical duration for 6 consecutive working days. There was a 6-day therapy break (4 working days and 1 weekend) between the two treatment intervals. Clinical assessment was carried out at 3 time points: before the commencement of the intervention (T1), after the first/before the second therapy interval (T2), and after the second interval (T3); see Figure 2.

The training materials consisted of card sets with matching picture cards in each set; these were specifically designed for the present study. To ensure comparability between the two treatment groups and between the two therapy methods and to exclude pure repetition effects, card sets were counterbalanced across groups and treatments. Importantly, therapy materials did not overlap with any of the test items included in the clinical language tests. Each card set consisted of 12 picture pairs with varying difficulty levels, including 48 different pictures, each of them with corresponding concrete words of high, medium, and low normalized lemma frequency; phonological minimal pairs; and items from only 1 semantic category. All card sets were split into 2 packets with equal numbers of items per difficulty level and assigned to ILAT or naming therapy in counterbalanced order across treatment groups. More detailed information has been reported recently.^26^ A group setting with 3 patients and 1 therapist was applied during ILAT and INT. The same therapist conducted all therapy sessions. All 18 patients completed the clinical trial as well as all pre-treatment and post-treatment assessments. Therapy sessions took place at an outpatient rehabilitation center and clinical assessments at the Brain Language Laboratory, Free University of Berlin.

### Intensive Language-Action Therapy

Language skills were practiced with behaviorally relevant communicative speech acts used in everyday life contexts. This was done by using card sets depicting objects differing in word frequency. As in previous ILAT studies,^17-22^ barriers on the table were used to prevent players from seeing other players’ cards and to facilitate verbal communication. Speech acts and complexity of communicative interactions were adjusted to patients’ therapeutic needs, verbal skills, and progress. The therapist served as a role model and engaged in purposeful communications while using positive reinforcement and shaping as behavioral methods throughout the therapy. Patients were encouraged to use spoken language.^17^

### Intensive Naming Therapy

INT resembled ILAT in as many ways as possible. The main difference was that verbal utterances in INT were not used with a pragmatic, communicative goal, but only to practice naming and describing objects. Thus, during INT, patients and the therapist did not engage in behaviorally relevant communicative speech acts used in everyday life contexts. However, identical to ILAT, the difficulty level of the target words was adjusted to patients’ language skills and therapeutic needs. No barriers were used during INT. Patients took turns in naming and describing objects from their card stacks, so that each patient received the same amount of practice. Words and picture cards of similar type and difficulty were used as targets in both treatment methods, and importantly, the number of verbal utterances between the two methods did not differ.^26^

### Clinical Assessment

A clinical neuropsychologist blinded to the group assignment and not involved in recruitment or treatment conducted all assessments. The primary focus of our analysis was on the BDI, a standardized and easy-to-administer self-report inventory that measures characteristic attitudes and symptoms of depression.^33^ Importantly, the BDI is designed to assess the presence and severity of symptoms of depression according to the *Diagnostic and Statistical Manual of Mental Disorders, Fifth Edition* (DSM-5)^12^ criteria. The BDI has a high internal consistency, with Cronbach’s α coefficients of .81 and .86 for nonpsychiatric and psychiatric populations, respectively, and is one of the most widely used clinical tests to measure the severity of depressive symptoms.^33^

In validation studies,^8,34^ the BDI was identified to have good sensitivity and specificity consistent with the DSM^12^ criteria for major depression and has been used in previous research to assess depression in participants with aphasia.^5,10,35,36^ The present study used the BDI-5, a simplified 20-item German version of the original BDI.^37^ Items on the BDI-5 are rated on a 4-point Likert scale (0-3) indicating symptom severity and are summed up to a total BDI score ranging between 0 and 60. This test version has been validated with large clinical and nonclinical cohorts; higher scores on the BDI indicate more severe symptoms of depression (for more details on classification of symptom severity see Table 1). Because language comprehension was relatively unimpaired in most patients, administration of the BDI was unproblematic. When necessary, patients were guided by the blinded clinician while filling out the BDI, for example, by reading out loud the statements or by rephrasing them. For 3 patients with global aphasia, BDI ratings in all testing sessions were additionally confirmed by their partners.

To assess language ability in the clinical context, we used the AAT,^29^ a standardized aphasia test battery with good test-retest reliability. Four subscales of the AAT assessing speech production and language comprehension skills were used: the token test, repetition, naming, and auditory comprehension. We excluded the AAT subscale “spontaneous speech” because of its partly insufficient construct validity and the subtest “written language” because the focus in both treatments was on spoken language. AAT results were designated as normally distributed T-scores, averaged across subscales.

Both treatment methods focused on training of verbal output and expression; therefore, the AAT subscales requiring speech production—naming and repetition—were considered as the most relevant subscales in the analyses. Mean AAT scores, which served as the primary outcome measure, have previously been reported in detail.^26^ Briefly, the analysis of mean AAT scores demonstrated significant improvements only with ILAT, legitimizing further exploration of changes in a secondary outcome measure, the BDI (for scoring details, see Table 1), related to different language interventions.

### Statistical Analysis

T-tests were carried out for each dependent measure to assess pre-treatment baseline differences between groups. No significant between-group differences at baseline were found for the variables age, education level, years after disease onset, memory, ADL, AAT, and BDI, confirming that the randomization process did not result in any pre-treatment group differences. Both groups were comparable with regard to clinical diagnosis and sex (see Table 1).

A repeated-measures analysis of covariance, ANCOVA, based on BDI scores was calculated with the between-subject factor Group (1 and 2) and the within-subject factor Time (T1, T2, T3). One-tailed *P* values and α levels of .05 were applied for all statistical tests. To assess treatment-induced changes in BDI scores irrespective of changes in language performance after each therapy interval, AAT scores were included as a covariate in this analysis. More specifically, the following covariates were included: (1) progress in language performance across the entire therapy phase (T3-T1) on the AAT subscale repetition, (2) progress in language performance across the entire therapy phase (T3-T1) on the AAT subscale naming, and (3) average baseline performance on the AAT. The latter factor was chosen because it is a measure of aphasia severity and the other two factors because they best revealed language-related therapy progress in this study.^26^

## Results

### Aachen Aphasia Test

Descriptive statistics, calculated as mean T-scores derived as averages from the four AAT subtests revealed the following means and SDs (in square brackets) for both groups and 3 assessment times: group 1 (T1: 49.4 [9.4], T2: 52.5 [9.5], T3: 52.1 [10.8]) and group 2 (T1: 48.7 [6.7], T2: 50.3 [7.0], T3: 52.9 [7.5]). Higher T-values indicate better language abilities.

A repeated-measures ANOVA revealed a significant interaction of Time and Group based on AAT mean scores [*F*(2, 30) = 6.91; *P* = .002; η^2^ = 0.12], again, calculated as mean T-score values from the four AAT subtests. The following ANOVA contrasts revealed better language performance after ILAT than after INT after both intervals [Time × Group interaction between T_1_ and T_2_: *F*(1, 15) = 4.72; *P* = .046; η^2^ = 0.08]. This pattern of results was even more pronounced when focusing on AAT speech production scores.^26^

Language performance (mean AAT scores) was used as a covariate in the ANCOVA analysis that was calculated for changes in BDI scores after each treatment interval.

### Beck Depression Inventory

Descriptive statistics were calculated based on mean BDI scores (SDs are in square brackets) for both groups and 3 assessment times: group 1 (T1: 17 [10.5], T2: 9.6 [9], T3: 10 [7.9]) and group 2 (T1: 25.7 [19.4], T2: 21.6 [15.6], T3: 17.1 [13.5]). Higher BDI scores indicate more severe symptoms of depression.

**Figure 3.**
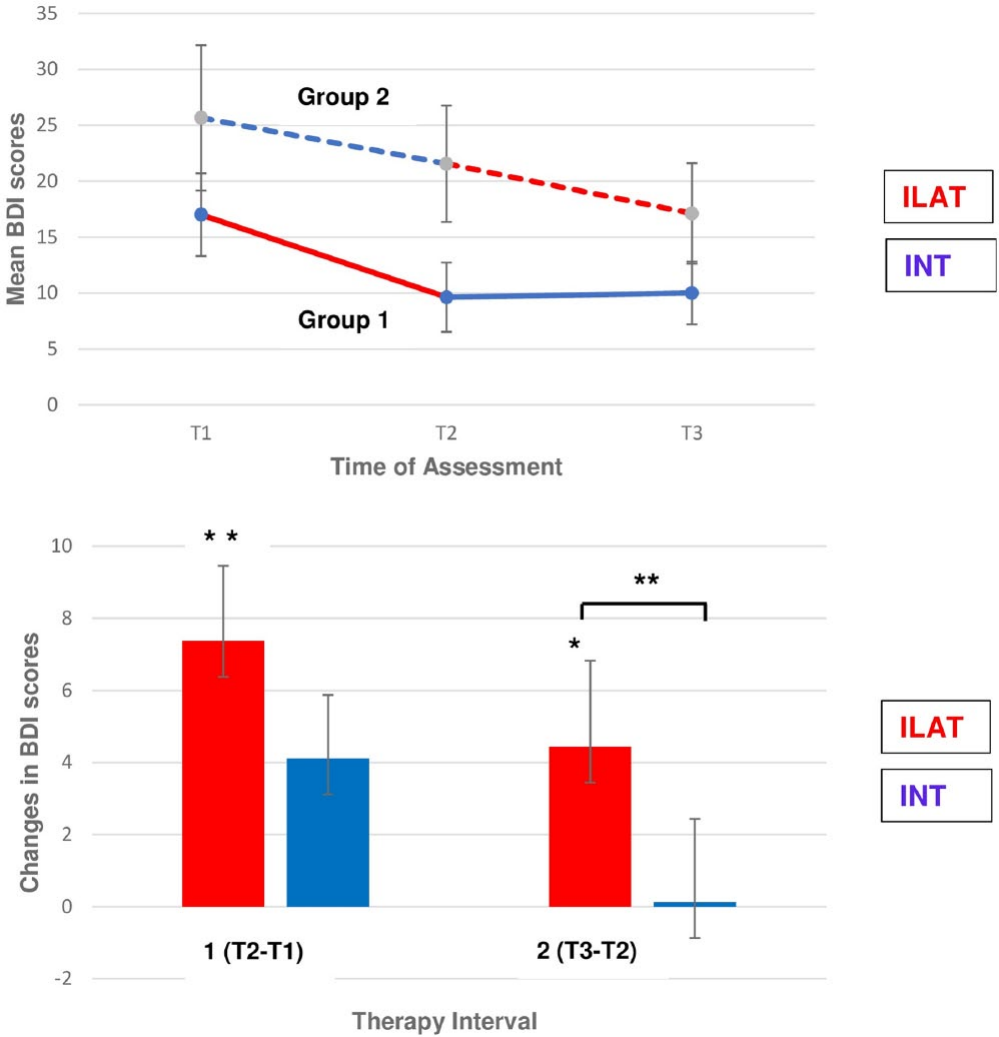
Changes in symptoms of depression, as assessed by the Beck Depression Inventory (BDI), across language therapy in chronic aphasia. A. (Top) The significant Group × Time interaction is displayed. Mean pre-therapy and post-therapy BDI scores for each group, treatment method, and therapy interval are presented: intensive language-action therapy (ILAT) in red; intensive naming therapy (INT) in blue. Higher BDI scores indicate more severe symptoms of depression. B. (Bottom) Reductions in BDI scores within therapy interval 1 (T2 − T1) and 2 (T3 − T2), pooled for both groups, are displayed separately for both treatment methods: ILAT in red and INT in blue. Significant post hoc differences are indicated by asterisks (*P < .05; **P < .01).

A repeated-measures ANCOVA on BDI mean scores revealed a significant interaction of Group and Time [*F*(2, 24) = 3.34; *P* = .026; η^2^ = 0.13], demonstrating a medium effect size. The interaction emerged primarily from the final training interval, where patients receiving ILAT improved on the BDI as opposed to those patients who received INT [Time × Group interaction contrast between T_2_ and T_3_: *F*(1, 12) = 12.37; *P* = .002; η^2^ = 0.32)]. In contrast, the interaction was absent in the initial training interval [Time × Group interaction contrast between T1 and T2: *F*(1, 12) = 1.44, *P* = .13, not significant; see Figure 3A]. Wilcoxon signed-rank tests showed significant BDI improvements after ILAT in the first (*z* = 2.52; *P* = .006) and second therapy intervals (*z* = 1.90; *P* = .03). In contrast, there was only a marginal improvement after INT in the first (*z* = 1.41; *P* = .08) and no change in the second treatment interval (*z* = 0.21; *P* = .42). These differences between therapy methods are displayed in Figure 3B. Symptom improvement across ILAT intervals was consistently observed, whereas no significant improvement in symptoms of depression occurred after treatment with INT. The statistical significance of any result reported here did not change when excluding the 2 patients with aphasia not caused by stroke: *F*(2, 20) = 3.35; *P* = .036; η^2^ = 0.13.

## Discussion

This study aimed to explore the potential effect of two different, intensive, language rehabilitation programs on language and symptoms of depression in patients with chronic aphasia, a frequently neglected patient population in the study of PSD. The results clearly showed that intensive communicative language therapy has a beneficial effect not only on language but, crucially, on symptoms of depression in chronic aphasia. This is the first RCT in participants with aphasia that demonstrates the efficacy of a specific, nonpharmacological treatment in reducing symptoms of depression in chronic patients. Our results document a statistically significant decrease in symptoms of depression specifically after treatment with a highly effective aphasia therapy regime, ILAT, emphasizing communication in social interactions. The treatment-related changes in BDI scores after ILAT intervals in both groups indicate clinically relevant changes in severity of depressive symptoms specifically for this aphasia therapy. Based on studies assessing the minimally important clinical difference (MCID)^38^ on the BDI-II in patients with major depression, it has been suggested that pre-post BDI changes of 5 or higher^39^ or a decrease in BDI scores of 17.5% or higher^38^ can be interpreted as clinically relevant. In our study, ILAT-related reductions in mean BDI scores (pooled across both groups) were 27%, whereas INT led to a 9.8% reduction, which was statistically nonsignificant. According to Button et al,^38^ the decrease in BDI scores after ILAT can be interpreted as a MCID and was independent of clinical improvements in language performance, as assessed by subscales on the AAT. As shown by our previous work,^26^ changes in expressive language ability in chronic participants with aphasia are likewise stronger with this intensive communication method (ILAT) than with intensive naming (INT), although the coexisting improvement in symptoms of depression cannot be explained by language restitution.

All patients in this study were at the chronic stage of the disease; therefore, spontaneous fluctuations in language abilities and mood are unlikely.^40^ Any behavioral changes observed can, therefore, be attributed with some confidence to the short-term intensive therapies. Furthermore, the fact that patients did not significantly benefit from INT suggests that the group setting and nonverbal social interactions per se cannot explain the reduction in depression scores because these aspects were comparable across both treatments. Moreover, treatment effects cannot be attributed to medication because this was not changed during the study. Notably, the number of depressed and nondepressed patients (see Table 1) receiving antidepressant medication did not differ between the two treatment groups.

It may be a strength of our study that we assessed symptoms of depression in a clinically relatively homogeneous sample of the nonfluent aphasia type, which is commonly associated with depression.^4^ However, we note that our results cannot be generalized to patients with fluent aphasia or to subacute or acute patients; this will have to be tested in future studies. Besides the higher prevalence of depression in nonfluent aphasia,^4^ the advantage of focusing on nonfluent patients is also their relatively good comprehension abilities, making it possible to obtain self-report depression ratings when questions are guided by a clinician.

The average age of our patient sample was slightly less than that of the general stroke population; nevertheless, it was comparable to previous studies on aphasia rehabilitation.^21,22^ It should be noted that the average age of ischemic stroke is steadily decreasing because of a significantly higher incidence of stroke in younger individuals (<50 years) in recent years.^41^ The high prevalence rate of poor functional outcome associated with affective and psychosocial problems in this patient group requires optimization of neurorehabilitation treatment. Apart from this trend toward stroke at younger age, on the positive side, younger patients are often more motivated and willing to participate in neurorehabilitation than older patients. This enhanced motivation in younger patients might constitute a selection bias, generally, in all kinds of rehabilitation studies. Moreover, many patients in our study were recruited from aphasia self-help groups, which could also have contributed to a selection bias because these groups are usually frequented by motivated patients. Finally, our sample size was relatively small and the exclusion of the one patient without aphasia according to AAT Token Test criteria slightly reduced our initial statistical power. In future, it would be desirable for further RCTs to include a larger number of patients while keeping the homogeneity of the group with regard to clinical variables.

Much effort was spent to balance the two treatments for unspecific features, such as attention, training intensity, stimulus materials, or group size and setting (see Methods). It cannot be completely ruled out that unspecific effects associated with the therapeutic process per se (e.g, getting more attention, being more active) have contributed to the improvement in depressive symptoms in our sample; however, this would equally apply to both treatment methods. Therefore, the beneficial effects of ILAT are unlikely to have emerged from unspecific factors but may more realistically be attributable to specific features of the training methods applied.

Patients in group 2 showed a trend toward reduced BDI scores after the first training interval when INT was applied, but this change was statistically not significant. Group 1 did not show any change in symptom severity after INT during the second therapy interval, which could potentially be attributed to a ceiling effect. However, the significant decrease in BDI scores in group 2 between T2 and T3 and the absence of such a decrease in group 1 during this time interval may indeed indicate that ILAT is more effective than INT.

Which features of ILAT could make this method effective in reducing symptoms of depression? ILAT is a treatment method derived from neuroscientific principles, including massed practice, focus on verbal expression, and crucially, behavioral relevance, including the use of spoken language in communication and social interaction contexts. Our study isolated the latter property; the distinct feature of ILAT as compared with INT was the behaviorally relevant and action-embedded use of verbal utterances^17^ in the context of social group interactions. Whereas the practice of naming in the INT context focused on specific target words, whose production was likewise an aim of ILAT, the flexible use and the broader repertoire of speech acts and communicative goals in ILAT may have been relevant features. Accordingly, the action-related and social aspects of communicating about objects to be passed from one player to the other often leads to positive interpersonal interactions, which may have contributed to mood improvements and to a reduction in depressive symptoms. Although future research is necessary to prove a causal link, our present results suggest a treatment-specific positive effect of ILAT, particularly of its behaviorally relevant communicative and social interaction component, on symptoms of depression. This effect was observed when controlling for individual changes in language performance, making it unlikely that reduced BDI scores arise directly from changes in language skills, as assessed by clinical language tests, and the associated positive assessment of own behavior. Still, the possibility exists that, during ILAT, patients may have improved communicative-pragmatic skills not assessed by clinical language tests, which, in turn, could have positively influenced patients’ symptoms of depression. Alternatively, aspects of the intensive communicative interaction implemented in ILAT may have an effect on symptoms of depression directly, without the mediation of language. Future research will be necessary to investigate any possible role of the specific features of language and communication therapy on symptoms of depression in chronic aphasia patients.

## Conclusions

This study demonstrates that ILAT, an effective aphasia therapy method, may help reduce symptoms of depression in patients with chronic nonfluent aphasia, a patient group at high risk of developing PSD. ILAT offers a short-term treatment that is suitable for brain-damaged individuals with impaired language and communication abilities, a clinical group that usually does not have access to nonpharmacological treatment of depression. Thus, intervention with intensive, behaviorally relevant communicative therapy and associated social interactions not only helps improve functional recovery of language, but may also enhance psychological well-being. In future, more studies are needed to investigate long-term stability of treatment effects as well as further variables that might be relevant for reducing depressive symptoms, such as the amount and duration of treatment. This will ultimately help improve the quality of life of patients with chronic aphasia.
